# Optimizing functional brain network analysis by incorporating nonlinear factors and frequency band selection with machine learning models

**DOI:** 10.1097/MD.0000000000041667

**Published:** 2025-02-28

**Authors:** Kaixing Hu, Baohua Zhong, Renjie Tian, Jiaming Yao

**Affiliations:** a School of Information Science and Engineering, Chongqing Jiaotong University, Chongqing, China; b School of Shipping and Naval Architecture, Chongqing Jiaotong University, Chongqing, China; c School of Mathematics and Statistics, Chongqing Jiaotong University, Chongqing, China.

**Keywords:** fCWT, functional brain networks, mild cognitive impairment, NMI, XGBoost

## Abstract

The accurate assessment of the brain’s functional network is seen as crucial for the understanding of complex relationships between different brain regions. Hidden information within different frequency bands, which is often overlooked by traditional linear correlation-based methods such as Pearson correlation (PC) and partial correlation, fails to be revealed, leading to the neglect of more intricate nonlinear factors. These limitations were aimed to be overcome in this study by the combination of fast continuous wavelet transform and normalized mutual information (NMI) to develop a novel approach. Original time-domain signals from resting-state functional magnetic resonance imaging were decomposed into different frequency domains using fast continuous wavelet transform, and adjacency matrices were constructed to enhance feature separation across brain regions. Both linear and nonlinear aspects between brain regions were comprehensively considered through the integration of complex correlation coefficient and NMI. The construction of functional brain networks was enabled by the adaptive selection of optimal frequency band combinations. The construction of the model was facilitated by feature extraction using tree models with extreme gradient boosting. It was demonstrated through comparative analysis that the method outperformed baseline methods such as PC and NMI, achieving an area under the curve of 0.9054. The introduction of nonlinear factors was found to increase precision by 14.25% and recall by 17.14%. Importantly, the approach optimized the original data without significantly altering the feature topology. Overall, this innovation advances the understanding of brain function, offering more accurate potential for future research and clinical applications.

## 1. Introduction

Cognitive decline has been shown to significantly impact quality of life, establishing brain health as a critical component of overall well-being.^[1]^ Alzheimer disease (AD), a chronic and irreversible neurodegenerative condition, is recognized as a major global public health challenge.^[2]^ Alterations in brain connectivity have been associated with various neurological disorders, including autism, attention deficit hyperactivity disorder (ADHD), and AD.^[3]^ Therefore, the study of functional brain connectivity is considered essential for understanding the mechanisms underlying these diseases. Functional brain connectivity is defined as the correlation of neuronal activity between different regions of the brain,^[4]^ and its analysis has been utilized to provide insights into cognitive impairments. Mild cognitive impairment (MCI), often regarded as a prodromal stage of AD,^[5]^ has been identified as a key focus of functional magnetic resonance imaging (fMRI) studies aimed at distinguishing MCI patients from normal controls (NCs).^[6–9]^

Many methods for constructing functional brain network (FBNs) have been proposed, with Pearson correlation (PC) and partial correlation (PCorr) being the most commonly used.^[8,10,11]^ Insights into the interactions and connectivity hubs of brain regions are provided by these methods.^[12]^ However, as they focus solely on linear relationships, the complex nonlinear dynamics within brain networks are often not captured.^[13]^ For instance, frequency-specific characteristics of fMRI signals have been observed across different brain regions.^[14,15]^ In cortical networks, connections are concentrated in the ultralow-frequency range (0.01–0.06 Hz), while the frequency range is broader (0.01–0.14 Hz) in limbic networks. High- and medium-frequency coherence can be observed in the limbic and temporal regions, but frontal connectivity is strongest at low frequencies (<0.08 Hz).^[14,15]^ Linear methods such as PC and PCorr inherently neglect these complex patterns, limiting their ability to fully capture brain dynamics.^[13]^

Efforts to address the nonlinear classification of FBNs have been explored. For example, FBNs were combined with support vector machine classifiers by Li et al to diagnose MCI,^[16]^ while time-constrained multiset canonical correlation analysis was employed by Wang et al to analyze blood oxygen level-dependent (BOLD) signals.^[17]^ The diagnostic accuracy of autism spectrum disorder (ASD) was significantly improved by Jie Yang et al by integrating functional brain networks with multiple connectivity patterns.^[18]^ However, linear and nonlinear relationships were rarely directly considered in these studies. Additionally, methods such as synchronous likelihood^[19]^ and spatial mutual information^[20,21]^ have been applied in other contexts, but they have either lacked efficiency or failed to generalize well across different frequency bands.

Recent studies have highlighted the importance of frequency-specific approaches for FBN analysis. The fast continuous wavelet transform (fCWT) has been identified as a promising tool for effectively decomposing fMRI signals into high-resolution time–frequency domains.^[22,23]^ Unlike traditional wavelet transforms, fCWT effectively captures correlations in both the time and frequency domains. Furthermore, normalized mutual information (NMI) has been utilized to identify complex nonlinear relationships that traditional methods, such as PC and PCorr, are unable to capture.^[24]^ The integration of fCWT and NMI has provided a powerful framework for revealing both intra-band and inter-band linear and nonlinear interactions.

Despite these advancements, limitations remain in the comprehensive analysis of nonlinear factors across different frequencies. Existing methods often rely on fixed frequency bands, posing a risk of missing critical interactions. Furthermore, many techniques neglect phase information, which is essential for accurately modeling the complex interactions between brain regions.^[13,23,25]^ To address these issues, a novel frequency-adaptive FBN construction method, combining fCWT and NMI, is proposed in this study. By adaptively selecting the optimal frequency bands, this method integrates linear and nonlinear characteristics to construct a comprehensive FBN. The main contributions of this paper are outlined as follows:

(1) A frequency-adaptive model based on fCWT is proposed. Higher accuracy and decomposition efficiency within the low-frequency band are demonstrated by this model compared to traditional wavelet transforms, with the optimal frequency combination with the best correlation being identified. This allows for the creation of FBN with different frequency combination responses.
(2) The enhanced frequency responses in the nonlinear factors between brain regions are investigated. By combining FBNs that represent both nonlinear and linear properties, classification tasks between MCI and NC are conducted, revealing a more comprehensive connectivity and interaction among brain regions.
(3) An important feature extraction method based on a tree model is adopted, with the nonlinear relationships between features being considered and the interactions among features being identified. This approach, which is suitable for the extreme gradient boosting (XGBoost) model, addresses the limitations of traditional linear feature extraction methods.

The remaining sections of this paper are structured as follows: the second section introduces the dataset, provides an overview of the data preprocessing methods, explains the basic principles of the correlation methods that are employed by these models, our methodology and model details are presented. The third section details the experimental setup. In the fourth section, the proposed method is comprehensively evaluated through experiments focused on MCI identification. The sensitivity of various parameters of complex correlation coefficient (CCC)-NMI is analyzed, as well as the significance of frequency responses and discriminative relationships. Finally, the paper concludes with a summary and insights into future research directions. To ensure reproducibility of the results, the preprocessed data and source code have been made available on GitHub.

## 2. Materials and methods

### 2.1. Data preparation

The dataset used in our experiments was sourced from the neuroimaging informatics tools and resources clearinghouse (NITRC), a publicly available repository. It included 46 participants with MCI and 45 NC, recruited through targeted advertisements in local newspapers and media. All participants were right-handed, had normal or corrected-to-normal vision, no history of neurological or psychiatric disorders, and reported no alcohol or drug abuse. Individuals undergoing regular medication treatment, taking psychotropic drugs, stimulants, or β-blockers were excluded from the study.^[26]^

Table 1 provides an overview of the demographic details for all subjects involved. The diagnosis of ASD was made based on the autism criteria outlined in the Diagnostic and Statistical Manual of Mental Disorders, Fourth Edition, Text Revision (DSM-IV-TR), while psychopathology assessments were conducted via parent or participant interviews to identify differential diagnoses and comorbidities-I disorders. Parent interviews utilized the Schedule for Affective Disorders and Schizophrenia for School-Age Children-Present and Lifetime Version (KSADS) for children under 17.9 years old. For participants over 18.0 years old, the Structured Clinical Interview for DSM-IV Axis-I Disorders, Non-patient Edition (SCID-I) and the Adult ADHD Clinical Diagnostic Scale (ACDS) were employed to determine psychopathology status. Exclusion from comorbid ADHD diagnoses was determined by meeting all criteria except criterion E within DSM-IV-TR guidelines, while inclusion as a neurotypical control required exclusion based on current KSADS-PL, SCID-I/NP, and ACDS interview results.

**Table 1:** Demographic information of the subjects.

	ASD (N = 45)	NC (N = 46)	*P*-value
Gender (M/F)	36/9	35/11	0.2135*
Age (year ± SD)	11.1 ± 2.3	11.0 ± 2.3	0.7773†
FIQ (mean ± SD)	106.8 ± 17.4	13.3 ± 14.1	0.0510
ADI-R (mean ± SD)	32.2 ± 14.3‡	–	–
ADOS (mean ± SD)	13.7 ± 5.0	–	–

ADI-R = autism diagnostic interview-revised, ADOS = autism diagnostic observation schedule, ASD = autism spectrum disorder, FIQ = full intelligence quotient, NC = normal control.

* The *P*-value in our analysis was calculated using the chi-squared test.

† The *P*-value in our analysis was calculated using the 2-sample 2-tailed *t* test.

‡ Two patients do not have the ADI-R score.

The original fMRI images were acquired using a standard clinical whole-body 3T MRI scanner from Siemens Trio in Germany, employing echo-planar imaging sequences. Participants were instructed to relax and focus their gaze on a white fixation cross displayed against a black background projected onto a screen throughout the scanning session. The imaging parameters were as follows: acquisition matrix size of 74 × 74, 45 slices, voxel size of 2.97 × 2.97 × 3 mm³, echo time of 30 ms, repetition time (TR) of 3000 ms, and a total of 180 repetitions. The preprocessing of the original fMRI images was conducted using statistical parametric mapping and Data Processing Assistant for Resting-State fMRI (DPARSF) (version 2.2) toolboxes, following established preprocessing protocols.^[27–29]^ To ensure signal stability, the initial 10 volumes of fMRI images from each participant were discarded. The remaining images underwent time correction and head motion correction across different slices.^[30]^ The remaining images underwent the following calibration steps: (i) standardization to the MNI space at a resolution of 3 × 3 × 3 mm^3^ (ii) nuisance signal regression, including ventricle, white matter, global signals, and head motion, using Friston 24-parameter model^[30,31]^; (iii) application of band-pass filtering within the frequency range of 0.01 to 0.08 Hz; (iv) signal detrending; (v) time points featuring frame shifts surpassing 0.5 were excluded to minimize the influence of minor head motion. Subsequently, the preprocessed BOLD time series signals were partitioned into 90 number of regions of interest (ROIs) based on the automated anatomical labeling atlas. Finally, these time series were organized into a data matrix, denoted as $X\in R^{80\times 91}$^[31]^.

### 2.2. Method

#### 2.2.1. fCWT

This method uses parameterized wavelet functions to process fMRI data, decomposing signals from the time domain into the time–frequency domain. The fCWT employs the Morlet wavelet as the mother wavelet function, and convolution operations are performed in the frequency domain. The Morlet wavelet is well-suited for signal processing and time–frequency analysis, which enables localized information to be provided along the time axis as well as frequency information across different scales. Subtle variations in time series signals are thus captured effectively.^[32]^ The formula for fCWT is presented as follows:


$$\begin{array}{l} fCWT(\alpha ,\tau )=F^{-1}\left[\begin{array}{l} F[x(t)]\cdot F[\psi \left(\frac{t-\tau }{\alpha }\right)]\; \end{array}\right] \end{array}$$

In the equation: the scale factor and translation factor of the wavelet transform are denoted by α and τ respectively; the input time signal is represented by x(t); the mother wavelet function is denoted by ψ(⋅); the Fourier transform is denoted by F; and the inverse Fourier transform is denoted by $F^{-1}$.^[26]^

The resting-state functional magnetic rs-fMRI time series data of participants are transformed into matrices $X\in R^{m\times n}$ after being processed. In this context, the number of time points in fMRI is represented by m, the ROIs in rs-fMRI data is denoted by n, and the time signal in fCWT is corresponded to by $x_{i}\in R^{m\times 1}$. Once the scale factor α, translation factor τ, and an appropriate wavelet function are set, the fCWT transformation is applied to all time points $x_{i}$, resulting in the original time signal being decomposed into signals at different frequencies:


$$\begin{array}{l} x(i)=fCWT_{\alpha_{k},\tau_{l}}(i)\in R^{a\times m} \end{array}$$

where: i=1,2,…,n;k=1,2,…,a;l=1,2,…,m.

#### 2.2.2. CCC

For the calculation of the CCC using the complex PC coefficient for the frequency domain decomposed signals x(i) obtained by fCWT, the formula is presented as follows:


$$\begin{array}{l} r_{c}=\frac{\sum_{i=1}^{n}\left(x_{i}-\bar{x}\right)(y_{i}^{*}-{\bar{y}}^{*})}{\sqrt{\sum_{i=1}^{n}|x_{i}-\bar{x}{|}^{2}\sum_{i=1}^{n}|y_{i}-\bar{y}{|}^{2}}} \end{array}$$

In the formula, $x_{i}$ and $y_{i}$ represent the i-th elements of complex signals x and y, respectively. $\bar{x}$ and $\bar{y}$ are the means of x and y, and $y_{i}^{*}$ denotes the complex conjugate of $y_{i}$.^[33]^ By incorporating the magnitudes and phases of complex signals into the PC coefficient, we can effectively assess the linear dependence between brain network signals decomposed by fCWT in the frequency domain. This approach reveals potential linear relationships and phase synchronization among brain regions, thereby capturing their functional connectivity.

#### 2.2.3. NMI

For each frequency-domain signal decomposed by fCWT, its MI is estimated. By using MI’s nonlinear patterns, the complex interactions of fMRI data from different brain regions under specific frequency decompositions are revealed. Unlike CCC, MI is focused more on nonlinear dependencies between different brain regions, providing a more accurate description of complex connectivity structures between brain regions. The formula for MI is presented as follows:


$$\begin{array}{l} I(X,Y)=\iint \hat{f}(x,y)\log\frac{\hat{f}(x,y)}{\hat{f}(x)\hat{f}(y)}dxdy \end{array}$$

In the formula: X and Y are sample data from 2 decomposed signals; $\hat{f}(x)$ is the probability density function of X, estimated using kernel density estimation with a smoothing kernel function.^[34]^

The formula for kernel density estimation is as follows:


$$\begin{array}{l} \hat{f}(x)=\frac{1}{Nh}\sum_{i=1}^{N}K\left(\frac{x-x_{i}}{h}\right) \end{array}$$

In the formula: K(⋅) is the kernel function, and h is the smoothing parameter. Then, construct the MI matrix by calculating the mutual information between all decomposed signals:


$$\begin{array}{l} M_{ij}=I(X_{i},X_{j}) \end{array}$$

By comparing the size of $M_{ij}$, the complex nonlinear relationships between the decomposed signals $X_{i}$ and $X_{j}$ can be captured.^[35]^ Finally, the MI matrix is normalized to maintain consistency of information across different signal scales, ensuring that matrix values are constrained between 0 and 1.

### 2.3. Model

Considering that varying responses across different frequency bands are exhibited by different brain regions, the construction of FBNs using fixed frequency bands may lead to erroneous connections. Additionally, more complex nonlinear relationships are often overlooked by correlation-based methods. Given that time series signals in both the time and frequency domains, particularly for low-frequency signals, are efficiently and accurately decomposed by fCWT, and complex nonlinear factors within sequences are captured by NMI, a new method is aimed to be developed by our study. This method integrates connectivity within the same frequency band and between different frequency bands, while considering both linear and nonlinear factors between brain regions. It aims to identify the most strongly correlated frequency sequences and achieve a more comprehensive understanding of interactions and associations between brain regions.

#### 2.3.1. Model overview

**Figure 1.**
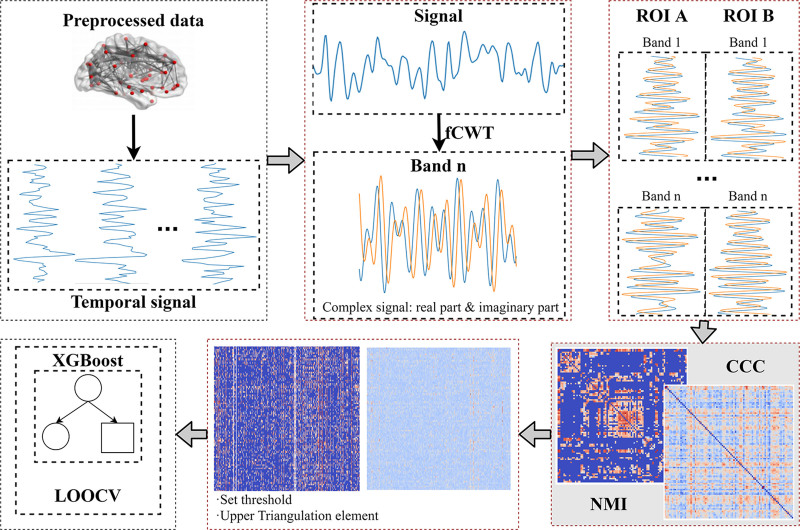
Workflow of our model.

To achieve a comprehensive and accurate understanding of the interactions and connections between brain regions, and for the construction of a FBN for classifying MCI and NC, 5 key steps are followed by our method upon the obtaining of the time series signal data. The fundamental concepts and primary steps of our model are illustrated in Figure 1.

Considering that the TR of the NITRC data is 3000 ms, the sampling frequency is approximately 0.33 Hz. The input parameters for fCWT include signal data, sampling frequency ($f_{s}$), starting frequency ($f_{0}$), ending frequency ($f_{1}$), number of scales ($f_{n}$), and the wavelet function. The parameter $f_{s}$ is required to be an integer. The Morlet wavelet is used as the default wavelet function. Details of the settings for other parameters are provided in Section 3.1. Therefore, the time series signal for each sample is resampled to update the sampling frequency to 1 Hz, meeting the requirements for the use of the fCWT. Subsequently, the fCWT is used to process the resampled signal.

For each brain region and frequency band, the CCC and NMI values are calculated for every possible frequency band combination.

Based on the results from the third step, the frequency band combinations with the highest correlation between different brain regions are adaptively selected to reconstruct the original time series signals. Using these selected frequency bands, the corresponding FBN is then constructed based on the CCC values.

A threshold is set for the FBN to construct a more representative network. The upper triangular elements are extracted to facilitate subsequent feature extraction tasks.

FBNs constructed with CCC and NMI under different thresholds are combined to comprehensively consider both linear and nonlinear factors. Important features are extracted based on a tree model and used in the XGBoost model for classifying MCI and NC.

#### 2.3.2. fCWT decomposition stage

For a signal series with a sampling frequency updated to 1 Hz, the i-th sample of the c-th column representing a region of interest is denoted as $X\left(i,c\right)=x_{c}^{i}\in R^{m\times 1}$, i=1,2,…; c=1,2,…n. According to equation 7, the fCWT is applied to obtain the decomposed components of this column’s region of interest, which are denoted as $fCWT_{\alpha_{k},\tau_{l}}\left(i,c\right)$. For simplicity in notation, this can be expressed as:


$$\begin{array}{l} {\left[\;fCWT_{\alpha_{k},\tau_{l}}\left(i,c\right)\right]}^{T}=\left[w_{1}^{\left(i,c\right)},w_{2}^{\left(i,c\right)},\ldots w_{a}^{\left(i,c\right)}\right]\in R^{m\times a} \end{array}$$

$$\begin{array}{l} w_{k}^{\left(i,c\right)}={\left(\;fCWT_{\alpha_{k},\tau_{1}}\left(i,c\right),fCWT_{\alpha_{k},\tau_{2}}\left(i,c\right),\ldots ,fCWT_{\alpha_{k},\tau_{m}}\left(i,c\right)\right)}^{T}\in R^{m\times 1} \end{array}$$

where k=1,2,…,a, with a representing the wavelet scales determined in the previous section, and m denoting the number of time points after resampling following the rs-fMRI data collection. Specifically, by decomposing each ROI of the first subject using the wavelet scale corresponding to the minimum frequency, we can construct a new matrix $X_{new}^{\left(1\right)}=\left(w_{1}^{\left(1,1\right)},w_{1}^{\left(1,2\right)},\ldots ,w_{1}^{\left(1,n\right)}\in R^{m\times n}\right)$, where n denotes the number of ROIs^[26]^.

For each permutation, a series of matrices $X_{new}$ can be generated. The objective is to identify the matrix $W_{new}$ that corresponds to the most pronounced correlation by identifying the matrix with the maximum absolute CCC or NMI values across different frequency combinations.

#### 2.3.3. Construct FBN

Based on the different frequency bands obtained from fCWT decomposition, the CCC and NMI between different brain regions and frequency bands within each sample are computed to derive matrix $W_{new}$. The calculations are limited to $\left(n\times \frac{\left(n-1\right)}{2}\right)\cdot \left(a\times \frac{\left(a-1\right)}{2}\right)$ times, with the focus solely on the upper triangle to minimize resource consumption. It is ensured by C that the frequency combination with the highest CCC and NMI values across all band pairs is retained. This forms the basis for constructing the corresponding FBN for the respective method.

Monte Carlo simulations were employed to compute the *P*-value corresponding to CCC for assessing its statistical significance. Since NMI calculation is rooted in information theory, evaluating statistical significance using *P*-values is not deemed suitable. Connection coefficients with *P*-values greater than or equal to .05 were updated to 0, and various thresholds were employed to obtain a more representative FBN. This process can be represented as follows:


$$\begin{array}{l} w_{ij}=\left\{ \begin{array}{ll} & 0,p_{ij}>0.05\;\\ & w_{ij}\Theta \left(w_{ij}-v_{t}\right),others\; \end{array}\right. \end{array}$$

where, $w_{ij}$ denotes an element in $W_{new}$, where i and j represent the CCC or NMI values between different brain regions i-th and j-th frequency groups, i,j=1,2,…,a. Θ denotes the Heaviside step function, equal to 0 for negative values and 1 for positive values. $v_{t}$ represents different percentile thresholds, t=0,10,20,…90,99, where t indicates the size of the corresponding percentile threshold. We can obtain FBN matrices $W_{CCCf,t}^{FBN}$ and $W_{NMIf,t}^{FBN}$ under different threshold values.

#### 2.3.4. XGBoost classification stage

Based on FBNs derived from combination of CCC and fCWT methods (CCC-fCWT [CCCf]) and combination of NMI and fCWT methods (NMI-fCWT [NMIf]) under different thresholds, various threshold combinations are traversed to extract upper triangular elements from matrices $W_{CCCf,t}^{FBN}$ and $W_{NMIf,t}^{FBN}\in R^{n\times n}$. This reduction of feature dimensions further facilitates the extraction of features. The resulting upper triangular matrix is denoted as $W_{t_{1},t_{2}}^{tri}\in R^{91\times \left(n\times \frac{n}{2}\right)}$, where the threshold selections for CCCf and NMIf are represented by $t_{1}$ and $t_{2}$, respectively. Using matrix $W_{t_{1},t_{2}}^{tri}$, important features are extracted using tree-based models for constructing an XGBoost classification model. To mitigate potential overfitting risks, a combination of leave-one-out and nested cross-validation techniques (LOOCV) is employed for hyperparameter selection.

## 3. Experiment

### 3.1. Experimental setting

After preprocessing, the NITRC dataset was reduced to 80 time points. Additionally, the BOLD signals of each participant were segmented into 90 ROIs using the automated anatomical labeling template. Our method was applied to the data, and a comparison was made with PC and NMI methods.

For resting-state brain functional data, it is reflected by low-frequency oscillations in the range of 0.01 to 0.08 Hz that spontaneous neural activity patterns occur during rest. Due to an updated sampling frequency of the original signal sequences, specifically, the frequency range of the original signals from 0.01 Hz to 0.08 Hz is mapped to 0.03 Hz to 0.24 Hz, resulting in an increase of the number of time points from 80 to 240. Thus, $f_{0}$ is set to 0.03, $f_{1}$ is set to 0.24, and $f_{n}$ is set to 10, representing the number of frequency bands to be decomposed. After applying fCWT to obtain different frequency bands, the maximum CCC and NMI values calculated across the frequency bands for a pair of ROIs are used to determine the optimal frequency band combination for that pair of ROIs. This combination is then applied to CCC and NMI, resulting in the proposed methods CCCf and NMIf. The *P*-values for CCC are computed using Monte Carlo simulations with 1000 iterations. Additionally, thresholds are set to [0, 0.1, 0.2, ..., 0.9, 0.95, 0.99].

After the FBNs for each sample have been obtained using CCCf and NMIf, the subsequent task involves extracting features for the classification of MCI and NC. Considering the symmetry of FBNs, only the upper triangular elements of each sample need to be extracted as input features to reduce feature dimensionality. In addition, the upper triangular elements of CCCf and NMIf under different thresholds are combined to form combination of CCCf and NMIf methods (CCC–NMIf). Using tree-based models, important features are extracted to construct an XGBoost classification model. The default setting is to extract twenty-five important features to further reduce the feature dimensionality.

Finally, an XGBoost model for classification is constructed. Three factors are considered: Firstly, the aim is to assess the impact of modifying the original data. More complex classifiers involve intricate feature selection schemes, where improvements may be confounded by changes in the original data versus changes from more challenging-to-identify feature selections. Secondly, the limited size of the experimental dataset is taken into consideration. According to statistical learning theory, models with higher complexity are required to have larger sample sizes,^[36]^ which inherently introduces risks of overfitting. Thirdly, the specific impact of inter-regional brain interactions and correlations on classification performance is aimed to be evaluated. Importance scores for each feature are provided by XGBoost, aiding in understanding the model’s decision-making process.

To reduce the potential risk of overfitting, a combination of LOOCV was used for training and hyperparameter selection. In this approach, the training samples were divided into inner and outer folds. The inner fold was used to determine the optimal hyperparameters through LOOCV, while the outer fold evaluated the test set using the best hyperparameters determined by the inner loop. This method not only effectively selects the optimal hyperparameters but also ensures the model’s generalizability, reducing the risk of overfitting. XGBoost was chosen as the classification model, considering its suitability for handling nonlinear relationships and complex feature interactions within the dataset. For hyperparameter tuning, the hyperparameters of XGBoost were optimized using RandomizedSearchCV, with the following parameters: number of estimators: [100, 200, 300]; maximum tree depth: [3, 5, 7, 10]; learning rate: [0.01, 0.1, 0.2]; random subset ratio of training samples: [0.8, 0.9, 1.0]; feature sampling ratio per tree: [0.8, 0.9, 1.0]; minimum loss for node splitting: [0, 0.1, 0.2]. During each cross-validation, the training data were standardized to ensure features were on the same scale. Through random search, the optimal hyperparameter combination was identified, thereby improving model performance. Finally, the optimized XGBoost model was used for training and evaluation.

### 3.2. Performance metrics

The classification results of different methods are evaluated using common performance metrics, including accuracy, recall, and F1-score. These metrics can be estimated using the following formulas:


$$\begin{array}{l} Accuracy=\frac{TP+FN}{TP+FP+TN+FN} \end{array}$$

$$\begin{array}{l} Precision=\frac{TP}{TP+FP} \end{array}$$

$$\begin{array}{l} Recall=\frac{TP}{TP+FN} \end{array}$$

$$\begin{array}{l} F1-score=2\times \frac{Precision\times Recall}{Precision+Recall} \end{array}$$

Where TP, FP, TN, and FN respectively denote the numbers of true positives, false positives, true negatives, and false negatives. Additionally, we use the area under the curve (AUC) to represent the performance of classifiers based on the receiver operating characteristic (ROC) curve, enabling comparison of classification performance across different methods.

## 4. Results and discussion

### 4.1. Classification results

Four classic methods were selected to estimate FBN on the NITRC dataset: PC, a linear correlation-based method; NMI, a nonlinear correlation-based method; CCCf, using frequency-adaptive fCWT; and CCC-NMIf, which incorporates nonlinear influences from NMI into CCCf. Thresholds of 0.60 for PC, 0.70 for NMI, 0.00 for CCCf, and 0.10 for both thresholds in CCC-NMIf were used, as these values were found to yield optimal results for each method at the specified thresholds. Results are detailed in Table 2.

**Table 2:** Results of different function brain network estimation methods.

Method	Accuracy	Precision	Recall	F1-score	AUC
PC	60.47%	63.86%	53.33%	55.63%	0.6254
NMI	53.92%	52.52%	60.00%	55.07%	0.5188
CCCf	77.95%	79.78%	77.78%	78.26%	0.8499
CCC-NMIf	89.06%	87.82%	91.11%	89.20%	0.9054

AUC = area under the curve, CCCf = complex correlation coefficient with fast continuous wavelet transform, CCC-NMIf = combination of CCCf and NMIf methods, NMI = normalized mutual information, NMIf = normalized mutual information with fast continuous wavelet transform, PC = Pearson correlation.

According to Table 2, different frequencies of the original matrix were reorganized by CCCf and CCC-NMIf, achieving higher accuracy rates of 77.95% and 89.06%, respectively, compared to the baseline methods PC and NMI. Additionally, recall rates of 77.78% and 91.11% were demonstrated by CCCf and CCC-NMIf, surpassing those of PC and NMI. Optimal results for each method were achieved using thresholds set to 0.60 for PC, 0.70 for NMI, 0.00 for CCCf, and 0.10 for both CCC-NMIf measures. These findings indicate that the proposed approaches are better suited for detecting MCI and reducing the likelihood of false negatives.

Furthermore, a more comprehensive inter-regional interaction is incorporated by CCC-NMIf compared to CCCf, specifically integrating complex nonlinear correlations that are not considered by CCCf. An improvement of 14.25% in precision and a 17.14% improvement in recall compared to CCCf are shown by the results. This indicates that brain regions are not only connected linearly but also involve more complex interrelations and connections. Taken together, these factors are significantly enhanced in the effectiveness of our method in monitoring MCI.

**Figure 2.**
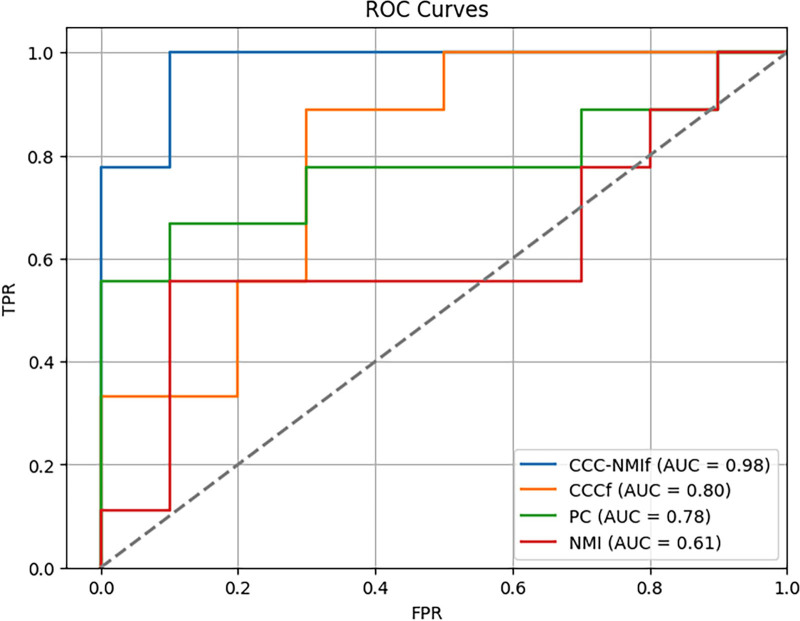
Receiver operating characteristic curves of PC, NMI, CCCf, and CCC-NMIf for classification performance. CCCf = complex correlation coefficient with fast continuous wavelet transform, CCC-NMIf = combination of CCCf and NMIf methods, NMI = normalized mutual information, PC = Pearson correlation.

To further evaluate the performance among different methods, the ROC curves and AUC values were compared. Figure 2 demonstrates that the ROC curve of CCCf closely resembles that of PC, indicating that CCC is suitable and accurate for handling correlations in complex signals. Modifications to the original data based on CCC were found to be successful. Additionally, a significantly higher AUC is exhibited by PC compared to NMI’s, confirming that traditional correlation-based methods are most suitable for estimating inter-regional connections in the brain. The highest AUC is achieved by CCC-NMIf, affirming that considering more comprehensive factors enhances accuracy, a point further supported by Table 2.

### 4.2. Comparison with state-of-the-arts

To benchmark existing methods for MCI classification, comparisons were made with 5 different approaches (Table 3), including: (1) AM-PC,^[37]^ which constructs FBNs with sparsity and low-rank constraints to identify important brain network regions; (2) QS-SAGCN,^[38]^ a quadruple Siamese network architecture combining graph convolutional networks with a self-attention mechanism; (3) MPGCN,^[31]^ a graph convolutional network based on information from different connectivity patterns; (4) 3D-CNN,^[39]^ a CNN classification model leveraging multidimensional features and Bayesian optimization; (5) HA-HI,^[40]^ a framework integrating fMRI and diffusion tensor imaging data based on dual-module hierarchical alignment and dual-domain hierarchical interaction modules.

**Table 3:** Comparison of the state-of-the-art methods for mild cognitive impairment classification.

Method	Accuracy	Recall	F1-score	AUC
1. AM-PC	0.8013	–	–	0.8773
2. QS-SAGCN	**0.935**	–	–	**0.922**
3. MPGCN	**0.911**	**0.910**	**0.916**	**0.971**
4. 3D-CNN	0.8077	–	0.8000	–
5. HA-HI	0.825	0.900	0.836	–
6. This work	0.8906	**0.9111**	**0.8920**	0.9054

The top 2 best results are shown in bold.

AUC = area under the curve.

From a performance perspective, MPGCN was found to outperform all other methods overall, while QS-SAGCN achieved the highest accuracy. Superior recall and F1-score were demonstrated by the proposed approach. AM-PC and 3D-CNN were observed to have relatively lower accuracy but remain reasonable baseline methods.

From an application scenario perspective, both the proposed method and AM-PC focus on optimizing FBN construction to identify important brain network regions, offering high biological relevance to brain network topology and making them suitable for FBN construction and analysis. QS-SAGCN and MPGCN were found to utilize GCNs, incorporating higher-order brain network information and multimodal connectivity data, making them well-suited for capturing complex higher-order topological information and inter-sample relationships. 3D-CNN employs fNIRS-based multidimensional features and automated hyperparameter optimization, requiring no manual intervention, which makes it ideal for community screening. HA-HI integrates regional connectivity features and employs SAM technology to enhance interpretability, making it suitable for precise analysis of brain networks in high-dimensional data environments.

### 4.3. Sensitivity analysis

**Figure 3.**
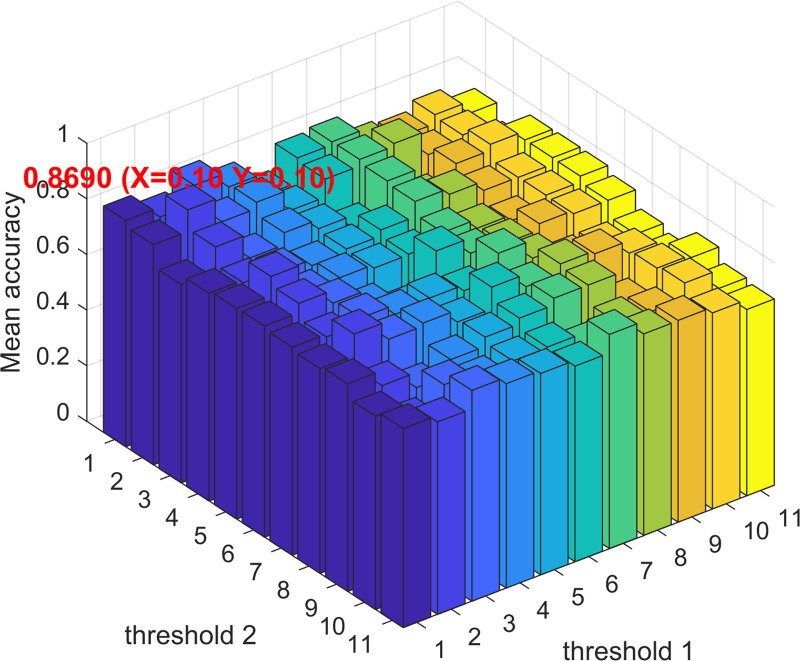
Average precision of CCC-NMIf under different threshold combinations. CCC-NMIf = combination of CCCf and NMIf methods.

It is noteworthy that the classification accuracy of the classic PC is quite sensitive to hyperparameter selection.^[29,41–43]^ To evaluate the sensitivity of CCC-NMIf to hyperparameters, a series of parameter experiments using different validation sets across various parameter ranges were conducted. Specifically, the threshold range was defined as [0.00, 0.10, 0.20,..., 0.90, 0.99], and the performance of the method under different hyperparameters was examined (Fig. 3). The results indicate that CCC-NMIf is less sensitive to parameter changes compared to PC,^[26]^ which can be attributed to the initial selection of important features through the tree model, reducing the dimensionality of the feature space and further improving the accuracy of FBN estimation. Notably, the highest accuracy (86.90%) was achieved by CCC-NMIf when both threshold 1 and threshold 2 were set at 0.10.

### 4.4. FBN estimation

**Figure 4.**
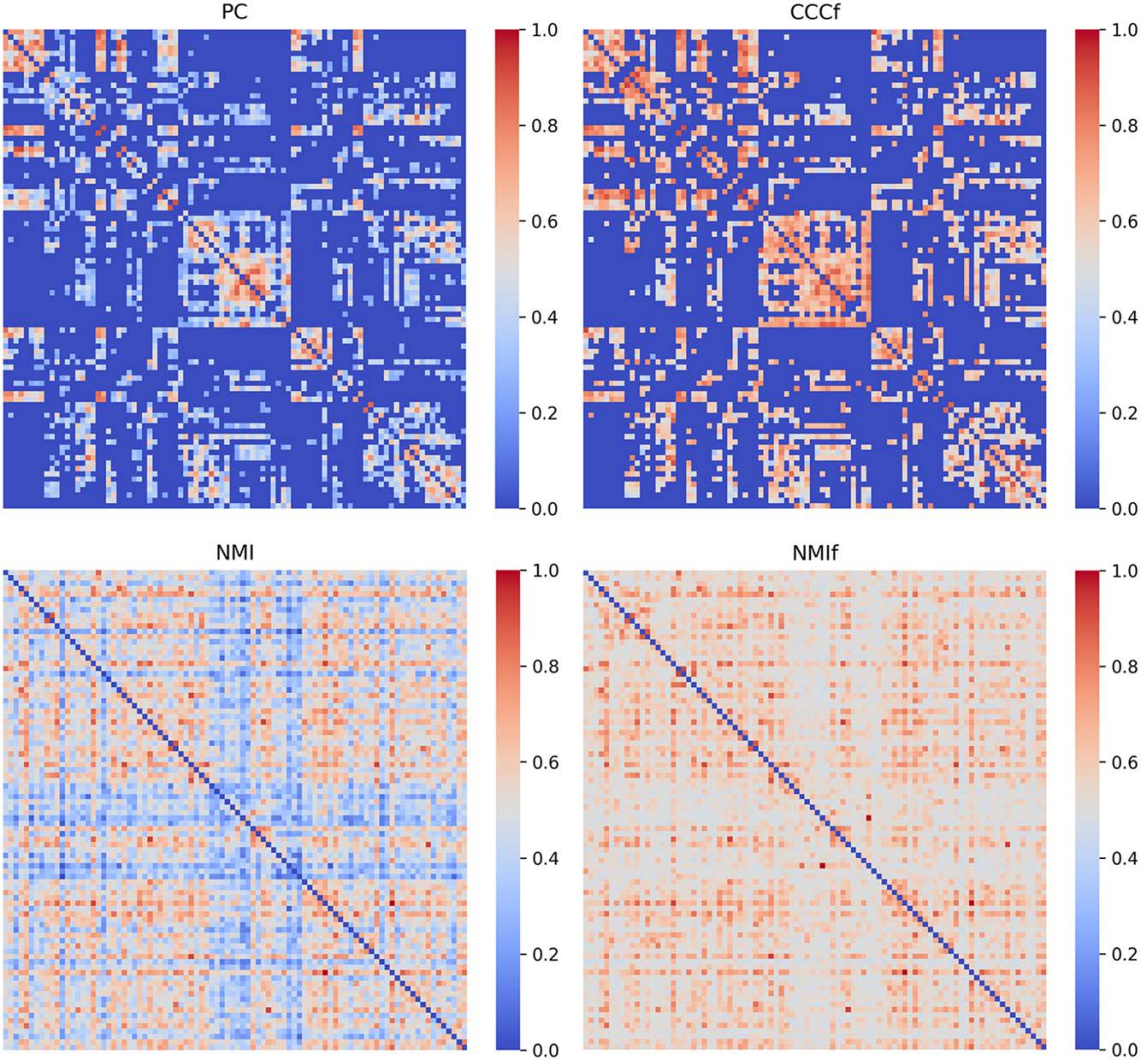
Weighted FBN networks using PC, CCCf, NMI, and NMIf methods. CCCf = complex correlation coefficient with fast continuous wavelet transform, NMI = normalized mutual information, NMIf = combination of NMI and fCWT methods, PC = Pearson correlation.

In this part, weighted FBNs for a selected subject are presented using PC, CCCf, NMI, and NMIf methods. To ensure comparability, all weights were normalized between 0 and 1. Differences in the FBN adjacency matrices generated by these methods on the NITRC dataset were explored, using a percentile threshold of 0 and applying a significance test with a *P*-value of <.05. Figure 4 demonstrates that similar structures are observed in NMI and NMIf, indicating minimal alterations to the topological structure of the original data by the method. The adjacency matrix processed by CCCf reduced many mixed-color regions. Specifically, the fully connected nature of PC resulted in its adjacency matrix being concentrated in strongly correlated regions while also including some low-correlation regions. This was reflected in the heatmap, leading to color mixing in certain areas, which may partially interfere with classification performance.^[26]^ In contrast, the adjacency matrices processed by CCCf and NMIf exhibit a more compact structure and deeper coloring in strongly correlated regions.

The connections involving the superior parietal lobule, angular gyrus, medial and paracingulate gyri, and superior temporal gyrus are focused on by both CCC-NMIf and CCCf (Tables 4 and 5). Notably, connections related to the hippocampus and parietal lobe, which are associated with MCI discrimination, are enhanced by CCC-NMIf through the incorporation of nonlinear factors.^[44–46]^ This phenomenon is significantly explained by parietal lobe-related connections, particularly those associated with the executive control network and salience network, with the supramarginal gyrus being shown to exhibit functional connectivity changes in MCI patients.^[47,48]^ Additionally, it has been indicated by studies that the progression from MCI to AD is associated with atrophy of the right caudate nucleus (CAU.R), with more significant atrophy in the right caudate nucleus being related to cognitive and memory impairment in MCI. The stronger functional connectivity in the right caudate nucleus (CAU.R) is also reflected by the nonlinear factors of NMIf.^[49,50]^ Unnecessary connections found in CCCf are eliminated by CCC-NMIf, effectively reducing the impact of weakly correlated connections, such as those involving the lentiform nucleus and gyrus rectus. This indicates that important features are highlighted and a comprehensive FBN construction is effectively facilitated by CCC-NMIf, through the incorporation of the NMI method. As shown in Table 4, the top 10 most discriminative connections identified by CCC-NMIf are mainly distributed in the frontal, temporal, parietal, and cuneus regions, corresponding to the default mode network, dorsal attention network, and fronto-parietal task control network.

**Table 4:** Top 10 most discriminative connections identified by the proposed method.

Connection		Feature contribution (10^-3^)	Resource
Region A	Region B		
PreCG.L	SMG.L	5.494	NMIf
SPG.R	IPL.R	5.347	CCCf
CAU.R	HES.R	4.783	CCCf
PCG.L	IPL.L	4.719	NMIf
MFG.R	DCG.L	4.703	NMIf
ORBinf.R	CUN.L	4.312	NMIf
ORBmid.L	SMG.L	4.255	CCCf
IOG.L	IOG.R	4.020	CCCf
ORBinf.L	HIP.L	3.981	NMIf
SOG.L	PUT.L	3.950	NMIf

CCCf = complex correlation coefficient with fast continuous wavelet transform, NMIf = normalized mutual information with fast continuous wavelet transform, feature contribution is expressed in 10^-3^ units.

**Table 5:** Top 10 most discriminative connections identified using the complex correlation coefficient with fast continuous wavelet transform.

Connection		Feature contribution (10^-3^)
Region A	Region B	
IFGoperc.R	IPL.R	9.654
SFGdor.R	ORBsup.L	8.059
SPG.L	IPL.L	7.122
SFGdor.R	PCL.L	6.806
ROL.L	HES.R	6.271
SFGmed.L	DCG.L	5.734
PUT.L	STG.L	5.097
SFGdor.R	PCL.R	4.935
REC.R	PAL.R	4.829
ORBsup.R	SPG.R	4.612

Feature contribution is expressed in 10^-3^ units.

### 4.5. Feature selection

**Figure 5.**
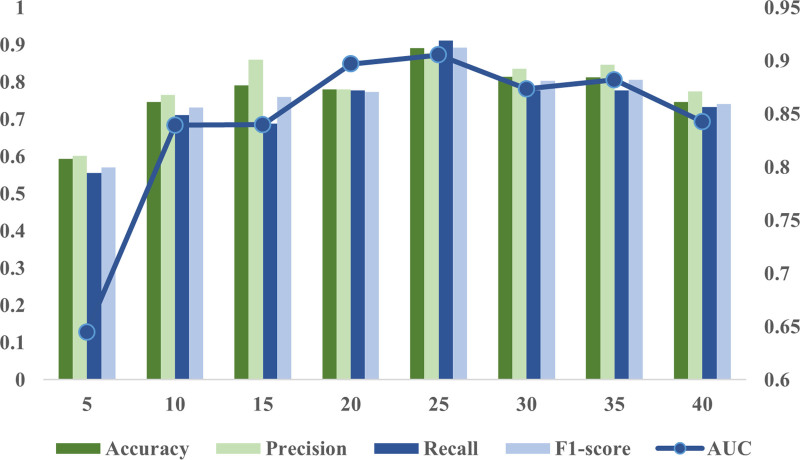
Effect under different numbers of important features selected.

In this section, the impact of the number of important feature extractions on the FBN estimation effect of the method is discussed. Optimal performance is reached by the method when the number of important feature extractions is 25 (Fig. 5). This further illustrates that in the classification task of MCI and NC, the understanding of the changes in more important connections is directly affected by the detection results. Feature extraction based on the tree model and the construction of a decision tree model are found suitable for this issue.

Furthermore, feature extraction based on the tree model is recognized as a necessary step to reduce the impact of insignificant features. Compared to the use of a two-sample t-test for feature selection and the construction of a simple linear SVM classification model,^[26]^ the feature extraction measures utilized in this method are found to be more suitable for tree models and for capturing nonlinear relationships. More precisely, the nonlinear relationships between features and the identification of interactions among features are captured by feature extraction based on the tree model, which are not addressed by the two-sample t-test. Given that more complex nonlinear relationships are considered by this method, the appropriateness of the feature extraction method using a tree model is affirmed.

### 4.6. Discriminative connections

**Figure 6.**
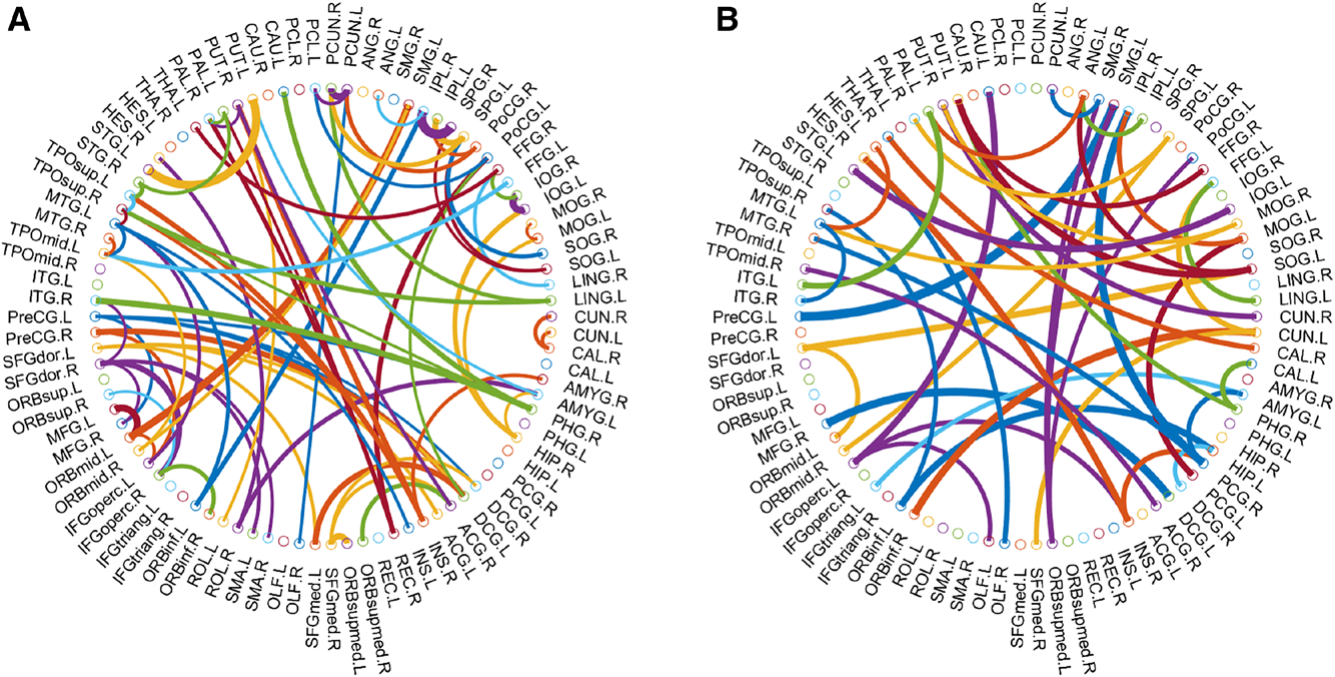
Most discriminative connection features. (a) Connection features for the CCCf part. (b) Connection features for the NMIf part. CCCf = complex correlation coefficient with fast continuous wavelet transform, NMIf = combination of NMI and fCWT methods.

Figure 6 illustrates the most discriminative connection features based on feature contribution, with arc thickness representing the discriminative ability of each connection in proportion to feature contribution. In Figure 6(A), the connection features for the CCC part in the CCC-NMIf method are shown, while in Figure 6(B), the connection features for the NMI part are presented. In constructing FBNs for classification from fMRI data, the examination of discriminative characteristics is considered critical. By considering both linear and nonlinear factors, features with high-resolution capacity can be pinpointed, aiding in the accurate identification of brain regions that significantly contribute to CCC-NMIf classification. The most discriminative connections were selected using feature extraction based on the tree model, as shown in Figure 6, through Paul Kassebaum “Circle Graph” function.^[51]^

The combined results of Table 4 and Table 5 are confirmed to show that for the linear relationship part of CCC-NMIf, the focus of the connections is mainly on the precentral gyrus, frontal lobe, temporal lobe, etc. For the more complex relationship part, the connections are complemented by those of the supramarginal gyrus, hippocampus, lingual gyrus, and superior occipital gyrus.

### 4.7. Important frequencies

**Figure 7.**
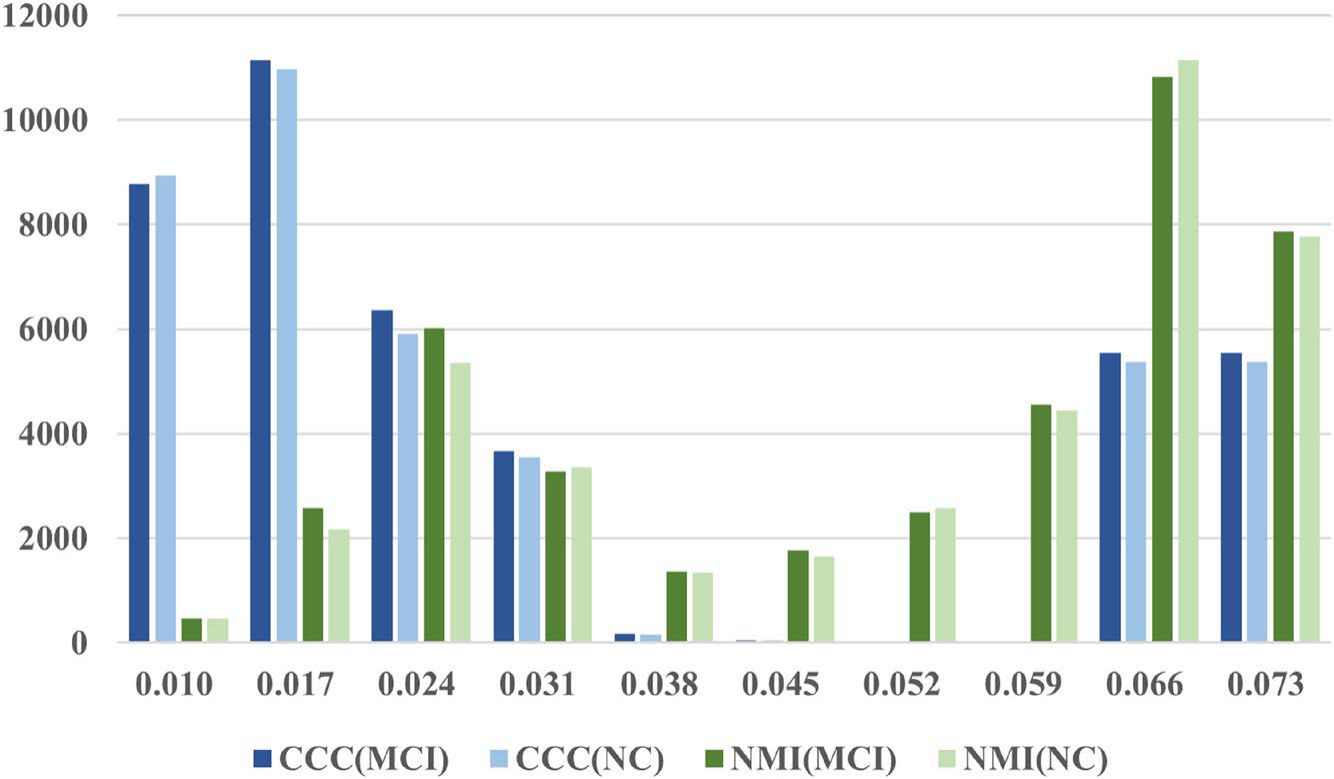
Frequency response relationships obtained by different methods.

Figure 7 illustrates the frequency response relationships obtained by different methods. The blue color is used to represent the number of response relationships at various frequencies as obtained through CCCf, while green is used for those obtained through NMIf. According to Figure 7 for MCI subjects, the second frequency band, representing a frequency signal of 0.017 Hz, is mainly highlighted by CCCf, whereas the ninth frequency band, representing a frequency signal of 0.066 Hz, is mainly highlighted by NMIf. The original signal is primarily decomposed in the frequency range of [0.01, 0.08]. In contrast, the low-frequency and high-frequency bands in linear relationships are primarily emphasized by CCCf, while all frequency band information is more comprehensively considered by NMIf in nonlinear relationships. Specifically, the frequency response level of MCI is elevated in linear relationships, while the response level in the high-frequency band is reduced in more complex relationships.^[52]^ In summary, the research results indicate that frequencies from 0.01 to 0.04 Hz are prioritized by CCCf, consistent with the previously mentioned sensorimotor, default mode, and visual cortical networks.^[14]^

## 5. Limitations and future work

To further improve the current classification method, several limitations need to be considered: (1) although the method is effective on the NITRC dataset, computational complexity is introduced by calculating CCC and NMI, as well as by using tree models for feature extraction in XGBoost. As the number of ROIs or frequency bands increases, challenges arise in its applicability to large-scale datasets or real-time scenarios. (2) While the method demonstrates scalability to high-dimensional data with numerous ROIs or time points, substantial computational resources and memory are required for practical deployment. (3) In the current study, only a single dataset was used. Further testing and validation across different datasets and clinical environments are needed to ensure robust generalization. (4) The limited data size in this study poses challenges for classification performance on larger datasets.

Future work could address these limitations by focusing on algorithm optimization to enhance scalability and model generalization, making the method applicable to broader samples and diverse clinical environments. Additionally, the application of fCWT and NMI techniques could be extended to multimodal neuroimaging data, such as structural MRI and diffusion tensor imaging, to provide more comprehensive brain network features, thereby further improving classification performance and expanding the applicability of the method.

## 6. Conclusion

A novel approach combining fCWT with NMI was successfully developed by this study, which significantly enhanced the classification accuracy between MCI and NC individuals. Using datasets from NITRC, a thorough analysis of rs-fMRI data was conducted by the research team. Time series signals were decomposed into different frequency bands using fCWT, and both CCC and NMI were computed to capture linear and nonlinear relationships between brain regions. By adaptively selecting optimal frequency band combinations for constructing FBN and employing an XGBoost model for classification tasks, superior performance over traditional PC and NMI methods was demonstrated by experimental results in terms of accuracy, precision, recall, and F1 score, achieving an AUC of 0.9054. Furthermore, the introduction of nonlinear factors was shown to increase precision by 14.25% and recall by 17.14% compared to linear methods, addressing significant gaps in linear connectivity. Additionally, the impact of different numbers of feature extractions on classification performance was explored by the study, and the most discriminative brain region connections were identified. These findings indicate that an integrated approach considering both linear and nonlinear factors among brain regions enhances the comprehensive understanding of interactions and correlations, thereby significantly improving the detection of MCI.

## Acknowledgments

We would like to express our gratitude to Associate Professors Zhang Lei from Chongqing Jiaotong University for their thoughtful feedback on the manuscript and language editing. We appreciate the time and effort contributed by all research participants.

## Author contributions

**Conceptualization:** Jiaming Yao.

**Data curation:** Kaixing Hu.

**Formal analysis:** Kaixing Hu.

**Funding acquisition:** Kaixing Hu.

**Investigation:** Kaixing Hu, Baohua Zhong, Renjie Tian.

**Methodology:** Baohua Zhong, Renjie Tian, Jiaming Yao.

**Project administration:** Kaixing Hu.

**Resources:** Kaixing Hu.

**Software:** Kaixing Hu, Jiaming Yao.

**Validation:** Baohua Zhong.

**Visualization:** Baohua Zhong.

**Writing – original draft:** Kaixing Hu.

**Writing – review & editing:** Baohua Zhong, Renjie Tian.
